# INKA2-AS1 Is a Potential Promising Prognostic-Related Biomarker and Correlated with Immune Infiltrates in Hepatocellular Carcinoma

**DOI:** 10.1155/2023/7057236

**Published:** 2023-05-02

**Authors:** Wenke Li, Guoqing Hong, Xing Lai

**Affiliations:** ^1^Department of Hepatobiliary Surgery, Yongchuan Hospital of Chongqing Medical University, Chongqing, China; ^2^Department of Hepatobiliary Surgery, Tongnan District People's Hospital, Chongqing, China

## Abstract

Hepatocellular carcinoma (HCC) is a malignancy with one of the worst prognoses. Long noncoding RNAs (lncRNAs) may be important in cancer development and may serve as new biomarkers for the diagnosis and treatment of various tumors, according to mounting research. The purpose of this study was to investigate the expression of INKA2-AS1 and clinical importance in HCC patients. The TCGA database was used to obtain the human tumor samples, while the TCGA and GTEx databases were used to gather the human normal samples. We screened differentially expressed genes (DEGs) between HCC and nontumor tissues. Investigations were made into the statistical significance and clinical significance of INKA2-AS1 expression. A single-sample gene set enrichment analysis (ssGSEA) was used to examine potential relationships between immune cell infiltration and INKA2-AS1 expression. In this investigation, we found that HCC specimens had considerably greater levels of INKA2-AS1 expression than nontumor specimens. When utilizing the TCGA datasets and the GTEx database, high INKA2-AS1 expression showed an AUC value for HCC of 0.817 (95% confidence interval: 0.779 to 0.855). Pan-cancer assays revealed that numerous tumor types had dysregulated levels of INKA2-AS1. Gender, histologic grade, and pathologic stage were all substantially correlated with high INKA2-AS1 expression. A survival study indicated that HCC patients with high INKA2-AS1 expression have shorter OS, DSS, and PFI than those with low INKA2-AS1 expression. Multivariate analysis indicated that INKA2-AS1 expression was an independent prognostic factor for OS of patients with HCC. According to immune analysis, the expression of INKA2-AS1 was favorably correlated with T helper cells, Th2 cells, macrophages, TFH, and NK CD56bright cells and negatively correlated with Th17 cells, pDC, cytotoxic cells, DC, Treg, Tgd, and Tcm. The results of this study collectively suggest that INKA2-AS1 has the potential to be a novel biomarker for predicting the prognosis of HCC patients as well as a significant immune response regulator in HCC.

## 1. Introduction

Liver cancer ranks sixth in terms of incidence among malignancies and is the fourth leading cause of tumor-related death worldwide [1, 2]. Over 782,000 individuals lose their lives to cancer annually, with over 841,000 new cases being diagnosed [3]. Hepatocellular carcinoma (HCC), the most prevalent kind of primary liver cancer, has been connected to several recognized risk factors, including a history of chronic HBV or HCV infection, excessive alcohol consumption, nonalcoholic fatty liver disease, and exposure to food toxins like aflatoxins [4, 5]. Even if a number of creative management strategies have demonstrated considerable effects on the diagnosis of HCC, the high rate of metastasis leads to poor overall survival (OS) of patients with HCC [6, 7]. The prognosis of patients is severely impacted by the fact that over 70% of HCC patients who have surgical resection or ablation will experience a tumor recurrence within five years [8, 9]. Accurately predicting the prognosis may help select an appropriate customized treatment and, as a result, increase the survival time for patients with HCC. The identification of novel biomarkers that can assess the prognosis of HCC cases is, therefore, crucial.

The term “long non-coding RNA” (lncRNA) refers to RNAs that are longer than 200 nucleotides yet cannot code for proteins [10]. In the past, lncRNAs were considered to be “transcriptional noise” since they did not take part in the process of creating proteins [11, 12]. As a result, it was believed that lncRNAs did not have any biological purpose. However, recent research has shown that lncRNAs have a biological function [13, 14]. The role of lncRNAs in several biological processes, including the silencing of X chromosome genes, chromatin modification, and transcription activity, has come under greater scrutiny in recent years [15, 16]. Recent research has identified a large number of lncRNAs as being improperly expressed in a variety of malignancies, which either inhibits the growth of these tumors or causes them to worsen [17, 18]. According to reports, several lncRNAs are crucial in the development of HCC [19, 20]. For instance, Hu et al. reported that in HCC, there was an increase in the level of expression of the lncRNA GSTM3TV2. The downregulation of lncRNA GSTM3TV2 via the miR-597/FOSL2 axis led to a considerable inhibition of cell proliferation and invasion [21]. When coupled with surrounding normal liver tissue samples and normal liver cell lines, Wang et al. found that the expression of the lncRNA MIR210HG was considerably greater in HCC tissue samples and cells [22]. Because of this, MIR210HG is a great marker for separating HCC tissues from normal tissues. Significant tumor growth, vascular invasion, an advanced clinical stage, and unfavorable histological differentiation have all been demonstrated to be related to high levels of MIR210HG expression. The survival research results showed that patients with high levels of MIR210HG expression had a considerably worse prognosis than patients with low levels of MIR210HG expression, both in their cohort and the TCGA cohort. HCC cells' ability to proliferate, migrate, and invade was decreased when MIR210HG was expressed less. These results indicated that novel diagnostic and prognostic lncRNAs for HCC patients hold substantial promise. On the other hand, it has not been investigated how many lncRNAs function. In this study, we discovered a new lncRNA called INKA2-AS1 that is associated with HCC and found that it is substantially expressed in HCC. Then, in addition to examining its connection to immune cell infiltration, we analyzed its diagnostic and prognostic relevance in more details. The findings of our study suggested that INKA2-AS1 may be a novel diagnostic and predictive biomarker for HCC patients, as well as a possible immune-related biomarker for HCC patients' treatment.

## 2. Methods

### 2.1. Microarray Data and RNA Sequencing Data

By clicking the URL (https://xenabrowser.net/datapages/), one can access the original mRNA expressions for the TCGA HCC data and GTEx from the UCSC XENA database. Human tumor samples came from the TCGA database, while normal human samples came from both the GTEx and TCGA databases. 374 liver cancer tissues and 160 healthy liver tissues collectively provided the data for the mRNA sequencing. 33 distinct cancers' RNA-seq transcriptome data were found by searching the TCGA database (https://portal.gdc.cancer.gov/). Included were the following 33 cancer types: ACC, BRCA, BLCA, COAD, ESCA, DLBC, HNSC, GBM, KICH, KIRC, KIRP, LGG, LIHC, LUAD, LAML, LUSC, OV, READ, PAAD, THCA, SKCM, UCEC, STAD, TGCT, THYM, PRAD, and UCS. We applied the Limma R package to screen the differentially expressed genes (DEGs) between HCC specimens and nontumor specimens. The cut-off value was determined to be log2FC greater than 2 and FDR less than 0.05 (FC, fold change; FDR, false discovery rate). It was not necessary for this study to get ethical approval or informed consent because the data on the TCGA databases are accessible to the general public.

### 2.2. General Enrichment Analysis

For the differential INKA2-AS1 obtained between single INKA2-AS1 high-/low-expression groups, additional GO enrichment analysis was performed. In addition, a KEGG signaling pathway analysis was carried out in order to determine which signaling pathways were engaged in the regulatory process. “ClusterProfiler” was used to carry out these two enrichment studies [23]. A false discovery rate (FDR) of 0.25 was chosen as the threshold for the statistical difference between the two enrichment analyses. Alternatively, the Metascape screening conditions were applied, and significant differences were defined by a *P* value of less than 0.05, a minimum count of 3, and an enrichment factor larger than 1.

### 2.3. Study of Immune Infiltration Using a Single-Sample Gene Set Enrichment Analysis (ssGSEA)

Using the ssGSEA method using the R package “sparcl,” 24 distinct kinds of immune cells discovered in tumor samples were examined for their immune infiltration of HCC [24]. The gene expression profile of each tumor sample was used to calculate the relative enrichment scores of each immune cell based on the marker genes of 24 distinct kinds of immune cells identified in the research literature. Both the correlation between immune cell infiltration and the groups with high and low levels of INKA2-AS1 expression and the correlation between immune cell infiltration and the groups with high and low levels of INKA2-AS1 expression were examined.

### 2.4. Statistical Analysis

Statistical analysis was performed using R (v.3.5.1) (R Core Team, 2018). Comparison of the expression of INKA2-AS1 between HCC specimens and nontumor specimens was carried out by the use of Wilcoxon rank sum tests. We separated patients into two groups: those whose gene expression was more than the median value and those whose gene expression was less than the median value. The relationship between clinical pathologic features and INKA2-AS1 was examined using the Kruskal-Wallis test or the Wilcoxon rank sum test in addition to logistic regression. The clinicopathological variables associated with 10-year overall survival (OS), progression-free interval (PFI), and disease-specific survival (DSS) in TCGA patients were identified using the Kaplan-Meier methods and Cox regression analysis. *P* values were two-sided, and a statistically significant difference was defined as one with a *P* value less than 0.05.

## 3. Results

### 3.1. INKA2-AS1 Expression Status in HCC Patients

**Figure 1:**
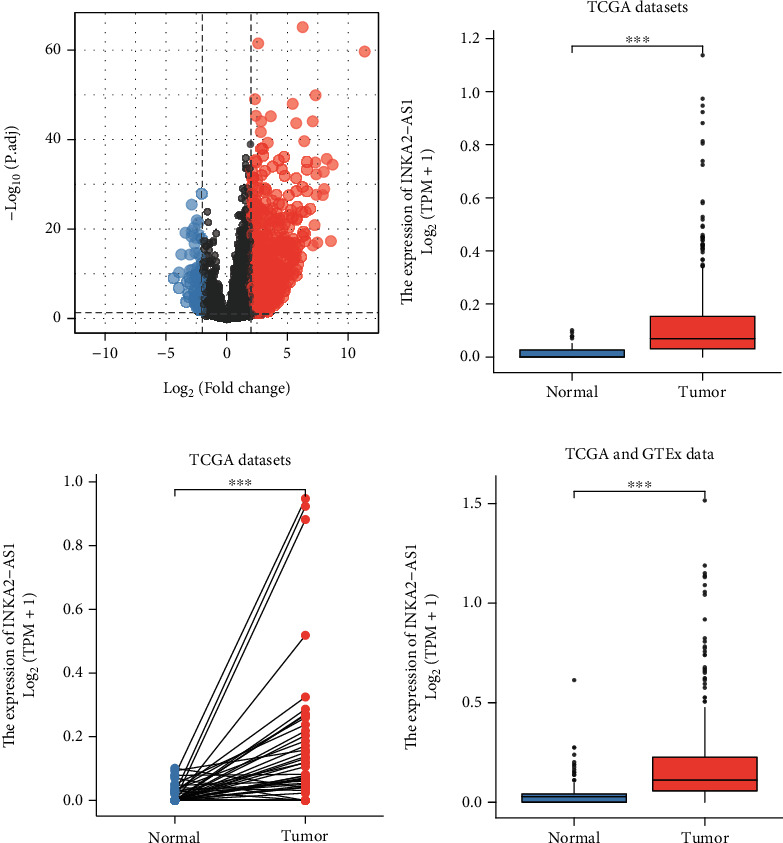
In HCC patients, INKA2-AS1 expression was noticeably elevated. (a) A volcano map displayed the DEGs between HCC and nontumor specimens. (b–d) The expression pattern of INKA2-AS1 in HCC specimens and nontumor specimens from the TCGA datasets or the TCGA datasets plus GTEx data.

In this work, information from 374 HCC and 50 nontumor specimens from TCGA databases was retrospectively examined. 938 DEGs include 859 strongly upregulated and 79 significantly downregulated genes (Figure 1(a)). Among the 938 DEGs, INKA2-AS1 caught our attention. We discovered that INKA2-AS1 expression was noticeably higher in HCC tissues compared to nontumor specimens, as illustrated in Figures 1(b)–1(d).

### 3.2. The Diagnostic Value of INKA2-AS1 Expression for HCC

**Figure 2:**
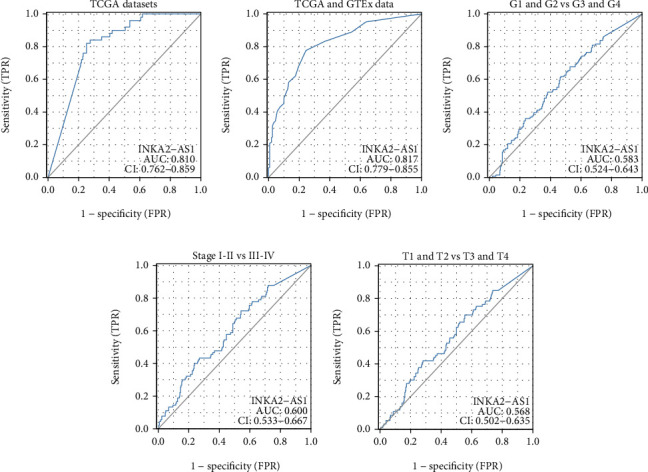
ROC analyses for the INKA2-AS1 expression in HCC's diagnostic value. (a, b) HCC specimens vs. normal specimens. (c) G1 and G2 vs. G3 and G4. (d) stage I-II vs. III-IV. (e) T1 and T2 vs. T3 and T4.

The diagnostic utility of INKA2-AS1 for patients with HCC was then investigated. Using the TCGA datasets, the ROC assays determined that HCC had high INKA2-AS1 expression, with an AUC value of 0.810 (95% CI: 0.762 to 0.859) (Figure 2(a)). The AUC value for high INKA2-AS1 expression in HCC was 0.817 (95% CI: 0.779 to 0.855) in the TCGA datasets and GTEx database (Figure 2(b)). Then, we performed subgroup assays, and the result is not ideal for histologic grade, pathologic stage, and T stage (Figures 2(c)–2(e)).

### 3.3. INKA2-AS1 Expression Analysis in Pan-Cancer

**Figure 3:**
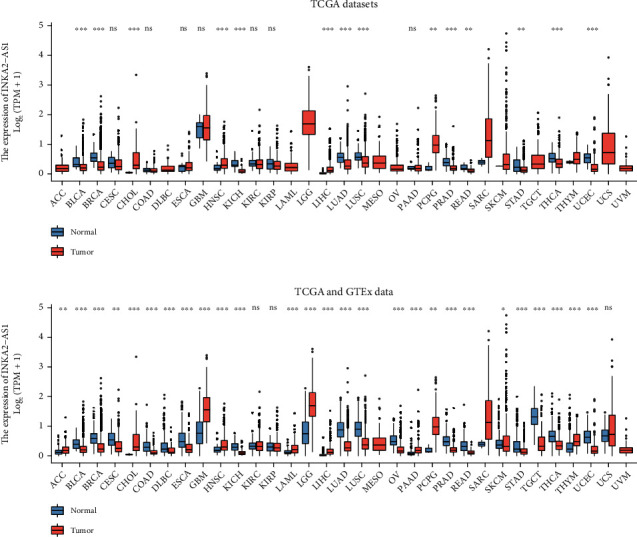
The expressions of INKA2-AS1 in various cancer types by analyzing (a) TCGA and (b) GTEx data and TCGA datasets.

Then, we examined the expression of INKA2-AS1 in the TCGA and GTEx databases. The findings indicated that high INKA2-AS1 expression was found in 14 tumors, including ACC, CHOL, GBM, HNSC, LAML, LGG, PAAD, PCPG, SARC, and THYM (Figures 3(a) and 3(b)). In contrast, low INKA2-AS1 expression was found in eleven tumors, including BLCA, COAD, BRCA, DLBC, CESC, KICH, ESCA, LUAD, LUSC, OV, and PRAD (Figures 3(a) and 3(b)). Our research indicated that INKA2-AS1 was crucial for the development of tumors.

### 3.4. Upregulation of INKA2-AS1 Associates with Advanced Clinicopathological Features of HCC

**Figure 4:**
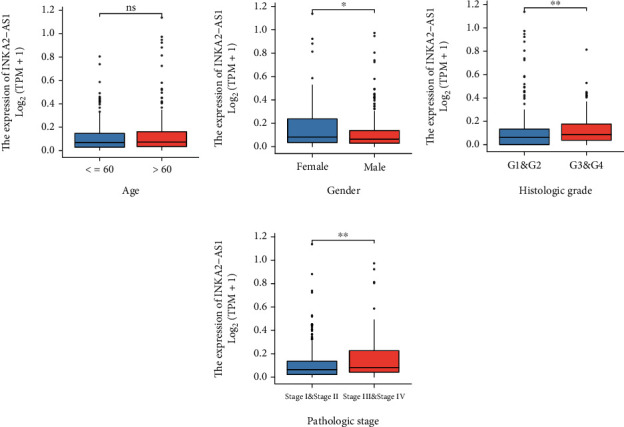
Relationship between clinicopathological traits such as (a) age, (b) gender, (c) histological grade, and (d) pathological stage and INKA2-AS1 expression.

We separated the 374 HCC patients into two groups based on the median INKA2-AS1 expression level, a high-expression group (*n* = 187) and a low-expression group (*n* = 187), to study further the clinicopathological importance of INKA2-AS1 levels in HCC patients. We observed that INKA2-AS1 expression was not related to age (Figure 4(a)) but was associated with gender, histologic grade, and pathologic stage (Figures 4(b)–4(d)). Additionally, the outcomes of the chi-square test supported the conclusions mentioned above (Table 1).

### 3.5. The Prognostic Value of INKA2-AS1 Expression in HCC Patients

**Figure 5:**
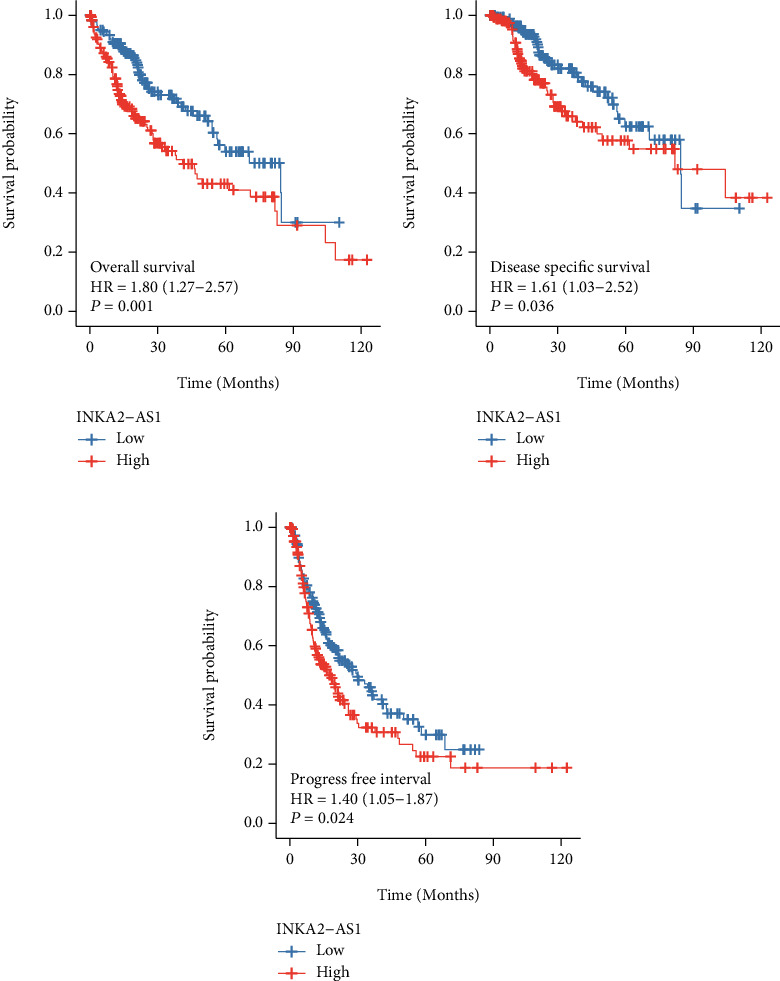
Kaplan-Meier survival curves contrasting HCC with high and low INKA2-AS1 expression. Survival curves of HCC patients with high and low INKA2-AS1 levels for (a) OS, (b) DSS, and (c) PFI.

We also performed a Kaplan-Meier analysis and a log-rank test to investigate the predictive significance of INKA2-AS1 expression in HCC. The findings revealed that HCC patients with high INKA2-AS1 expression had shorter OS (Figure 5(a), *P* = 0.001), DSS (Figure 5(b), *P* = 0.036), and PFI (Figure 5(c), *P* = 0.024) than those with low INKA2-AS1 expression. Then, we performed a Cox proportional hazard regression analysis. In patients with HCC, INKA2-AS1 expression was shown to be a standalone predictive factor for OS (Table 2, *P* = 0.005), according to multivariate analysis. However, neither the DSS (Table 3, *P* = 0.093) nor the PFI (Table 4, *P* = 0.075) of HCC patients showed evidence of INKA2-AS1 expression as an independent prognostic factor.

### 3.6. Functional Enrichment Analysis and DEG Profiles

**Figure 6:**
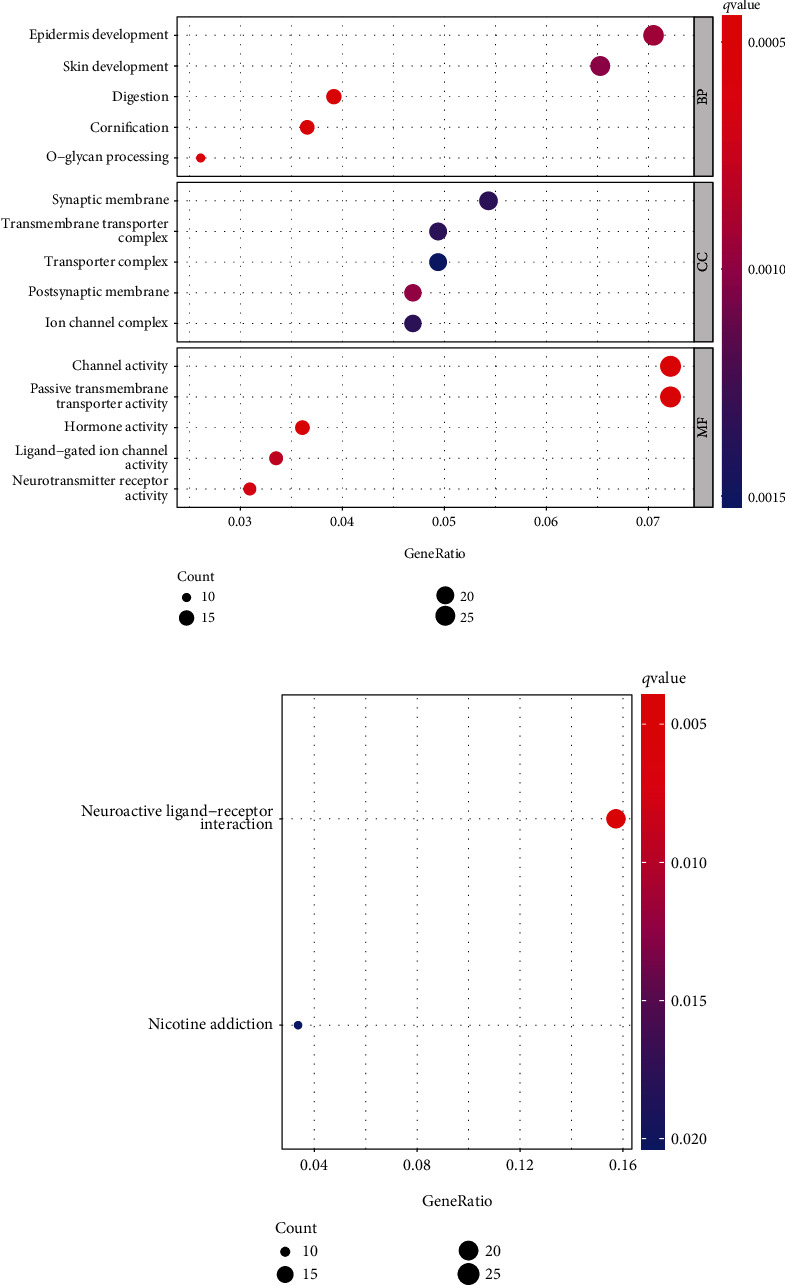
Functional enrichment analysis of DEGs comparing groups with high and low INKA2-AS1 expression levels. (a) DEGs with significantly enhanced GO terms. (b) Important KEGG pathway.

Based on the median value of INKA2-AS1 expression, we split the HCC patient population from the TCGA database into low- and high-expression groups to better understand the biological processes connected to the DEGs. There were 430 DEGs found in all. According to the findings of the GO tests, 430 DEGs were mostly linked to the growth of the epidermis, the skin, digestion, synapses, transmembrane transporters, channels, and hormone function (Figure 6(a)). According to KEGG assays, the primary enrichment areas for 430 DEGs were nicotine addiction and neuroactive ligand-receptor interaction (Figure 6(b)).

### 3.7. Immune Cell Invasion and INKA2-AS1 Expression Were Related

**Figure 7:**
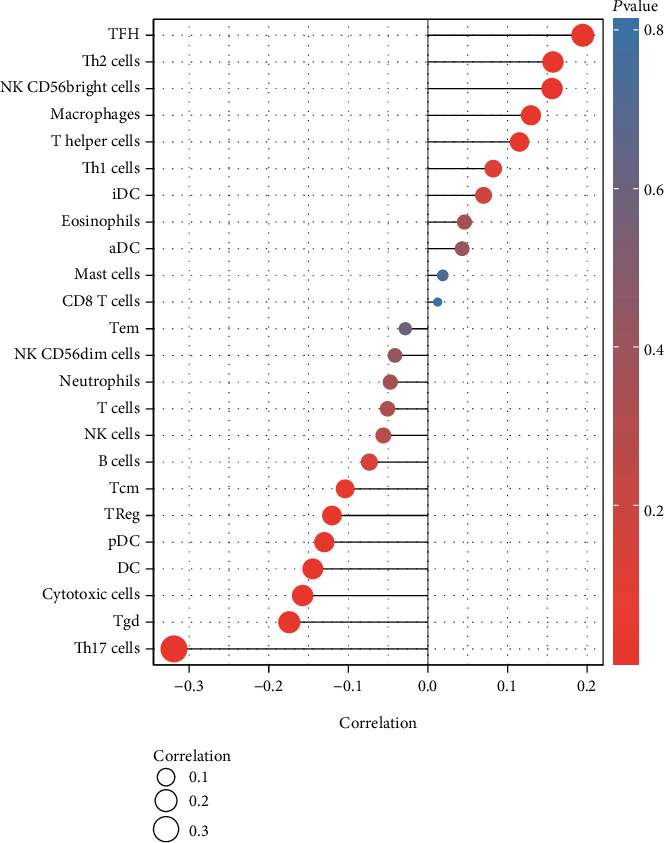
Immune cell infiltration was linked to INKA2-AS1 expression.

To ascertain the extent of immune cell infiltration, the transcriptomes of the TCGA HCC cohort were examined using the ssGSEA approach. The number of immune cells present in a tumor's microenvironment was quantified in the research using 24 immune-related genes. We found that the expression of INKA2-AS1 was favorably correlated with T helper cells, Th2 cells, macrophages, TFH, and NK CD56bright cells and negatively associated with Th17 cells, pDC, cytotoxic cells, DC, Treg, Tgd, and Tcm (Figure 7).

## 4. Discussion

HCC continues to be one of the most aggressive forms of solid malignancy found anywhere in the world [25]. The discovery of prognostic factors in HCC is critical for determining the most effective therapy options and predicting patients' survival rates [26]. To this point, a wide variety of biological markers have been reported. Growing studies have suggested that the abnormal expressions of antioncogene or tumor promotors played a vital role in the tumor growth and invasion of HCC [27, 28]. lncRNAs are the subject of increasing investigation as a possible new class of biomarker. In addition, several studies have discussed the potential of lncRNAs as predictive or diagnostic biomarkers for cancer [29, 30].

Several researches published in recent years have shown that lncRNAs have a role in the development of HCC and may serve as new biomarkers for HCC patients. For instance, Zhou et al. found that the expression of the lncRNA ID2-AS1 reduced in metastatic HCC cell lines and in HCC tissues. This lowered expression was associated with a poorer overall survival rate in HCC patients. lncRNA ID2-AS1 significantly reduced the motility, invasion, and metastasis of HCC cells in vitro and in vivo in HCC patients via activating the HDAC8/ID2 pathway [31]. Li et al. demonstrated that HCC tissues and HCC cells expressed the lncRNA DCST1-AS1 at a high levels. High expression of the lncRNA DCST1-AS1 was significantly correlated with a bad outcome. In addition, the absence of the lncRNA DCST1-AS1 led to a reduction in cell proliferation and an acceleration of apoptosis in HCC cells, as well as an activation of cycle arrest, a reduction in cell migration, and an increase in autophagy. These effects were mediated by the AKT and mTOR signaling pathways [32]. We discovered the HCC-related lncRNA INKA2-AS1 in this investigation. The expression of INKA2-AS1 was noticeably elevated in HCC patients, as we initially observed. In TCGA datasets, the diagnostic utility of INKA2-AS1 was also established. Furthermore, we discovered that poor prognosis and advanced stage were linked to increased expression of INKA2-AS1. It is significant to note that multivariate analysis revealed that INKA2-AS1 expression was a standalone predictive factor for OS in HCC patients. Our research indicated that INKA2-AS1 could be a new diagnostic and predictive biomarker for people with HCC.

Immunotherapy, which works by boosting patients' natural defenses against disease, has been successful in treating a variety of malignancies and converting them into illnesses that can be healed [33, 34]. Immune-based treatment methods have been shown to offer survival improvements for patients with HCC in a significant amount of preclinical and clinical researches [35, 36]. Additionally, it is envisaged that shortly a combination of immunotherapy and other therapeutic modalities may be a workable alternative for treating HCC. Additionally, recent studies have shown that tumor-infiltrating lymphocytes, including regulatory T cells and tumor-associated macrophages, play a critical part in the immune evasion that occurs during the progression of HCC [37, 38]. Because of the depletion of follicular helper T cells caused by intratumoral PDL1, poor B cell function was produced, which aided in the advancement of advanced HCC. In this work, we found that the expression of INKA2-AS1 was favorably correlated with T helper cells, Th2 cells, macrophages, TFH, and NK CD56bright cells and negatively associated with Th17 cells, pDC, cytotoxic cells, DC, Treg, Tgd, and Tcm. Our research revealed that INKA2-AS1 might be crucial for immune infiltrate cells to be recruited and regulated in HCC.

However, there are several limitations in our study. First, given the small sample size, thorough clinical testing will be required. Second, even though the bioinformatics study gave us some relevant insights of INKA2-AS1 in HCC, to validate our results and increase their therapeutic significance, we still need to perform biological research, either in vitro or in vivo. To further understand the function of INKA2-AS1 on both the molecular and cellular levels, additional research into its mechanistic studies is required.

## 5. Conclusion

We firstly provided evidence that demonstrated a considerably elevated expression level of INKA2-AS1 in HCC patients. As a predictive biomarker for HCC, INKA2-AS1 may function as a tumor promoter and predict prognosis as well as immune infiltration.

## Figures and Tables

**Table 1:** Correlation of INKA2-AS1 expression with clinicopathological features of HCC.

Characteristic	Low expression of INKA2-AS1	High expression of INKA2-AS1	*P*
*n*	187	187	
Age (*n*, %)			0.874
≤60	90 (24.1%)	87 (23.3%)	
>60	97 (26%)	99 (26.5%)	
Gender (*n*, %)			0.015
Female	49 (13.1%)	72 (19.3%)	
Male	138 (36.9%)	115 (30.7%)	
Histologic grade (*n*, %)			0.036
G1	36 (9.8%)	19 (5.1%)	
G2	91 (24.7%)	87 (23.6%)	
G3	52 (14.1%)	72 (19.5%)	
G4	6 (1.6%)	6 (1.6%)	
Vascular invasion (*n*, %)			0.217
No	113 (35.5%)	95 (29.9%)	
Yes	51 (16%)	59 (18.6%)	
Residual tumor (*n*, %)			0.220
R0	168 (48.7%)	159 (46.1%)	
R1	6 (1.7%)	11 (3.2%)	
R2	1 (0.3%)	0 (0%)	
Pathologic stage (*n*, %)			0.023
Stage I	100 (28.6%)	73 (20.9%)	
Stage II	37 (10.6%)	50 (14.3%)	
Stage III	35 (10%)	50 (14.3%)	
Stage IV	2 (0.6%)	3 (0.9%)	
T stage (*n*, %)			0.030
T1	105 (28.3%)	78 (21%)	
T2	39 (10.5%)	56 (15.1%)	
T3	35 (9.4%)	45 (12.1%)	
T4	5 (1.3%)	8 (2.2%)	
M stage (*n*, %)			1.000
M0	133 (48.9%)	135 (49.6%)	
M1	2 (0.7%)	2 (0.7%)	
N stage (*n*, %)			0.122
N0	129 (50%)	125 (48.4%)	
N1	0 (0%)	4 (1.6%)	
Age (median, IQR)	61 (52, 69)	62 (51, 68)	0.492

**Table 2:** Univariate and multivariate Cox regression analyses for overall survival.

Characteristics	Total (*N*)	Univariate analysis		Multivariate analysis	
		Hazard ratio (95% CI)	*P* value	Hazard ratio (95% CI)	*P* value
Gender	373				
Female	121	Reference			
Male	252	0.793 (0.557-1.130)	0.200		
Age	373				
≤60	177	Reference			
>60	196	1.205 (0.850-1.708)	0.295		
Histologic grade	368				
G1 and G2	233	Reference			
G3 and G4	135	1.091 (0.761-1.564)	0.636		
Pathologic stage	349				
Stage I and stage II	259	Reference			
Stage III and stage IV	90	2.504 (1.727-3.631)	<0.001	2.352 (1.618-3.419)	<0.001
INKA2-AS1	373				
Low	187	Reference			
High	186	1.804 (1.268-2.568)	0.001	1.722 (1.180-2.514)	0.005

**Table 3:** Univariate and multivariate Cox regression analyses for disease-specific survival.

Characteristics	Total (*N*)	Univariate analysis		Multivariate analysis	
		Hazard ratio (95% CI)	*P* value	Hazard ratio (95% CI)	*P* value
Gender	365				
Female	118	Reference			
Male	247	0.813 (0.516-1.281)	0.373		
Age	365				
≤60	174	Reference			
>60	191	0.846 (0.543-1.317)	0.458		
Histologic grade	360				
G1 and G2	227	Reference			
G3 and G4	133	1.086 (0.683-1.728)	0.726		
Pathologic stage	341				
Stage I and stage II	254	Reference			
Stage III and stage IV	87	3.803 (2.342-6.176)	<0.001	3.637 (2.233-5.924)	<0.001
INKA2-AS1	365				
Low	186	Reference			
High	179	1.612 (1.032-2.520)	0.036	1.529 (0.932-2.507)	0.093

**Table 4:** Univariate and multivariate Cox regression analyses for progression-free interval.

Characteristics	Total (*N*)	Univariate analysis		Multivariate analysis	
		Hazard ratio (95% CI)	*P* value	Hazard ratio (95% CI)	*P* value
Gender	373				
Female	121	Reference			
Male	252	0.982 (0.721-1.338)	0.909		
Age	373				
≤60	177	Reference			
>60	196	0.960 (0.718-1.284)	0.783		
Histologic grade	368				
G1 and G2	233	Reference			
G3 and G4	135	1.152 (0.853-1.557)	0.355		
Pathologic stage	349				
Stage I and stage II	259	Reference			
Stage III and stage IV	90	2.201 (1.591-3.046)	<0.001	2.123 (1.531-2.944)	<0.001
INKA2-AS1	373				
Low	187	Reference			
High	186	1.398 (1.045-1.870)	0.024	1.321 (0.972-1.795)	0.075

## Data Availability

The analyzed datasets generated during the study are available from the corresponding author on reasonable request.
